# A comprehensive bibliometric overview: antibiotic resistance and *Escherichia coli* in natural water

**DOI:** 10.1007/s11356-021-14084-1

**Published:** 2021-05-06

**Authors:** Semanur Şahin, Nüket Sivri, Isil Akpinar, Zeynep Birsu Çinçin, Vildan Zülal Sönmez

**Affiliations:** 1 Department of Genetics and Bioengineering, Nisantasi University, 34398 Istanbul, Turkey; 2 Department of Environmental Engineering, Istanbul University-Cerrahpasa, 34320 Istanbul, Turkey; 3 Department of Chemical and Environmental Engineering, University of Nottingham, Nottingham, NG7 2RD UK

**Keywords:** *Escherichia coli*, Antibiotic resistance, Agricultural and biological sciences, Network visualization, Publication analysis

## Abstract

The environment is the most important reservoir for both resistance mechanisms and gene transfer in biological science studies. This study gives a bibliometric overview of studies of “antibiotic resistance” and “*Escherichia coli*” in the field of “Agricultural and Biological Sciences” from 2015 to 2019 to assess both research trends and scholarly networks in diverse research disciplines. The two keywords of “antibiotic resistance” and “*Escherichia coli*” were selected to search in the Scopus database. Each review article was categorized into materials, natural waters (i.e., seawater, freshwater) and wastewater, journal name, and quartile in category of the journal, the year of publication, and the country. Bibliometric indicators and visualization maps were utilized to analyse the retrieved data quantitatively and qualitatively. A total of 1376 publications in the field of agricultural and biological sciences over the last 5 years were obtained using the keywords of antibiotic resistance and *Escherichia coli*. With additional keywords of freshwater and wastewater, 4 and 24 studies were obtained, respectively. Wastewater was found to be the most common working environment for the keywords of antibiotic resistance and *Escherichia coli*. It is also found that the studies of antibiotic resistance are mainly conducted in wastewater environments, focusing on human and food health. Working under “One Health” consisting of human, animal and agriculture, and environmental health could be the only permanent and effective approach to solving antibiotic resistance-related issues.

**Supplementary Information:**

The online version contains supplementary material available at 10.1007/s11356-021-14084-1.

## Introduction

Antibiotic was an important discovery which treats many infections and prevents the spread of infectious diseases. However, bacteria can rapidly evolve resistance to antibiotics (Pinheiro et al. 2020). In 2015, World Health Organization (WHO) established a Global Antimicrobial Resistance Surveillance System (GLASS) to act against antibiotic resistance (WHO 2015; WHO 2020a). Moreover, WHO published a global list of antibiotic-resistant bacteria based on their priorities. According to this list, twelve bacteria and bacterial families are classified into three categories as critical, high, and medium. Enterobacteriaceae, which is listed in the critical classification, is associated with carbapenem-resistant and third-generation cephalosporin-resistant (WHO 2017). A bibliometric analysis, in general, is performed to assess both research trends and scholarly networks in diverse research disciplines (Zhang et al. 2019). Keyword analysis identifies the most popular topics within the scope of bibliometric analysis (Ellegaard and Wallin 2015). In this study, the focus was on the research of antibiotic resistance and of *Escherichia coli* transmitted to human beings from the environment via direct or indirect routes such as consumption of the contaminated food and water. A bibliometric study can provide a significant dataset to determine the frequencies and deficiencies of the studies. These bibliometric analyses, therefore, provide solutions to the spread and prevention of antibiotic resistance in the environment.

It is known that wastewater can have an adverse effect on human, animal and agriculture, and environmental health (Sharma and Sharma 2021). Antibiotic resistance has been commonly studied in the wastewater and there must be a faecal reservoir to form an antibiotic resistance. In the priority list published by WHO, Enterobacteriaceae is considered as a priority bacterium group of antibiotic resistance (WHO 2017). A study reported that 28.3% of 353 isolates corresponded to Enterobacteriaceae in beta-lactamase production, while 83% of them was of *Escherichia coli* (Aristizabal-Hoyos et al. 2019). Therefore, it is found that *Escherichia coli* plays a significant role in the formation of resistance in aquatic ecosystems. As a result of examination of bibliometric analysis studies, it is seen that a number of studies focused on isolated *Escherichia coli* and their antibiotic resistance mechanisms conducted in in situ sampling in aquatic ecosystems are found to be insufficient. Monitoring antibiotic resistance in natural aquatic systems and the kind of antibiotics that are dominant play a significant role in ecosystems health. This study aims to provide preliminary information on studies on multi-drug–resistant *Escherichia coli*.

Bibliometric analysis is an important part of the evaluation method in applied fields. By examining various aspects of science in terms of its method, it offers a broad research analysis on the number of publications, the most productive countries, institutes, and authors, prominent journals, and the quartile of categories. This study provides a bibliometric analysis of the latest scientific studies on antibiotic resistance and *Escherichia coli* in the field of Agricultural and Biological Sciences from 2015 to 2019.

## Materials and methods

For this study, data search and collection were retrieved through the Scopus database. The search strategy encompassed the titles, abstracts, and keywords of the articles that were searched from 2015 to 2019. The document type was limited to articles; however, international presentations and book chapters were also found that are not considered in this study. Furthermore, the subject field was limited to the category of agricultural and biological sciences. The obtained data over the last 5 years from 2015 to 2019 were provided as title–abstract and keywords of “antibiotic resistance” and “*Escherichia coli*”, the publication year of 2015, 2016, 2017, 2018, and 2019 and the subject area of “Agricultural and Biological Sciences”. The information collected from the Scopus database was given as (i) country (country of study), (ii) antibiotic names which are given as multidrug (more than five drugs in the articles) or their names (less than five drugs in the articles), (iii) environment such as seawater, wastewater, and freshwater, (iv) journals’ name, (v) quartile of category, and (vi) other information.

Antibiotic resistance and *Escherichia coli* are the first search terms that were used to search the articles in the abstract or title in Scopus. The obtained data over the last 5 years from 2015 to 2019 were provided as title–abstract and keywords of “antibiotic resistance” and “*Escherichia coli*”, the publication year of 2015, 2016, 2017, 2018, and 2019, and the subject area of agricultural and biological sciences. In this study, title and abstract were searched separately using the keywords. Afterwards, the articles compared found by searching separately and published by the same authors and same title were accepted as one article. Using these two keywords, articles were classified according to them including *Escherichia coli* or antibiotic resistance or both. There are more keywords on resistance in the various topics encountered while searching the database; however, in this study, we focused on only antibiotic resistance. The terms “antibiotic resistance”, “antimicrobial resistance”, and “bacterial resistance” were used for *Escherichia coli* as well as the keywords of “coliform” and “Enterobacteriaceae” in the search in Scopus.

In this study, we analysed the studies of antibiotic resistance that were performed in the aquatic environment, but we did not analyse the studies that were done in other environments. The keywords of “freshwater”, “wastewater”, “seawater”, and “water” (other water environments) were used for the classification of water environments. Freshwater includes river, watershed, lake, waterways, groundwater, tap water, drinking water, and surface freshwater. Wastewater consists of sewage, sludge, environmental water, recycled water, drainage water, and reclaimed water. Seawater includes coastal, beach, marine, stream water, recreational water, and surface seawater. Where the publications do not have water media, they were grouped into animal, plant, human, soil, illness, food, and faeces. Soil was also added into this list where the soil is in contact with water. In the case of the publications, if they do not include any of these environments, these studies were classified as E (none). Furthermore, we searched for the country-based indicating the studies were conducted, or where the samples were taken. Where the country name was not presented in the abstract or title, the country names were presented as the country of the first author or corresponding author.

The drug names were presented as “multidrug” or their names of “Antibiotic1”, “Antibiotic2”, “Antibiotic3”, “Antibiotic4”, and “Antibiotic5” when the articles have more than five antibiotics or fewer than five antibiotics given in the studies, respectively. Moreover, if an antibiotic name was not included in the abstract or title, but it was mentioned in the study, this antibiotic was accepted as multidrug resistance. The summary or title may not contain an antibiotic name; however, it may contain a resistance gene, and therefore the antibiotic name was included in which the resistance gene is effective.

The bibliometric software plays a vital role in visualization of bibliometric analysis data. Distance-based and graphic-based mapping are being used in visualization of bibliometric analysis (Garfield 2009). While Kamada-Kawai, Fruchterman–Reingold, and Pathfinder techniques are being utilized for graphic-based mapping, multidimensional scaling, VOSviewer, VxOrd, and Kopcsa-Schiebel techniques are being used in distance-based mapping (Artsın 2020). In our study, VOSviewer was chosen to be utilized due to its ease of use.

## Results and discussion

This bibliometric analysis covers the studies performed up until 20 April 2020 and 1376 articles in 2015, 2016, 2017, 2018, and 2019 were found within the criteria searched in the Scopus database. It was found that “multidrug” is to be mostly preferred in antibiotic selection. The results obtained were interpreted in the headings of the most relevant journal names, publications, and quartile categories that contributed significantly to the research on antibiotic resistance, *Escherichia coli* and the environment (wastewater, seawater, freshwater, water). Also, it has been visualized with figures for clarity. It provides information that can be considered when submitting publications to the journal by providing data about the related area.

Quartiles are categorized into the four quartiles of Q1, Q2, Q3, and Q4. This means that Q1 is occupied by the top 25% of journals in the list, Q2 is occupied by journals in the 25 to 50% group, Q3 is occupied by journals in the 50 to 75% group, and Q4 is occupied by journals in the 75 to 100% group. The most prestigious journals within a subject area are those occupying the first quartile, Q1, and the importance of the other journals declines as we move down through the quartiles (MU Mondragon Unibersitatea 2020). It is found that the journal of “PlosOne” with 22 articles has the highest number and “Applied and Environmental Microbiology” has the second highest number of articles (11), and they are both ranked in the first quartile from 2015 to 2019. “Journal of Food Production” and “International Journal of Environmental Science and Technology” are in the second quartile and are found to be ranked top amongst the others in this quartile, having 5 and 4 articles, respectively.

**Fig. 1:**
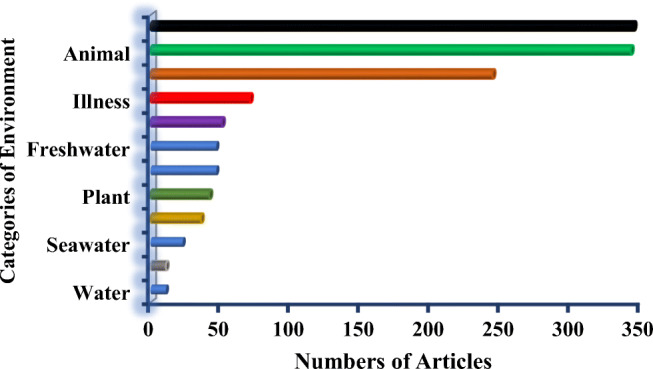
Antibiotic resistance and *Escherichia coli* studies with the number of articles published were performed in different categories of the environment in the field of agricultural and biological sciences from 2015 to 2019

Figure 1 shows the number of articles of antibiotic resistance and *Escherichia coli* studies in different categories. It can be seen that using the keywords of antibiotic resistance and *Escherichia coli* in the field of Agricultural and Biological Sciences are classified into different categories as E (none), animal, and food having a cumulative percentage of 45 according to the data of agricultural and biological sciences as expected. Overall, the numbers of articles published in each of the environment categories are E (none) (345 articles), animal (343 articles), food (244 articles), illness (71 articles), and human (51 articles) are given. On the other hand, wastewater (47 articles), freshwater (47 articles), plant (42 articles), faeces (36 articles), seawater (23 articles), water (11articles), and soil (11 articles).

From the results obtained by criteria considered in this bibliometric analysis, the first six leading countries in terms of the highest number of publications on antibiotic resistance and *Escherichia coli* studies were presented. The USA (24 articles), China (8 articles), and India (7 articles) are found to be the leading countries in the studies of antibiotic resistance and *Escherichia coli* in water environment. Nigeria is the fourth country on this list having 6 articles. Bangladesh and South Africa were both found to have 5 articles. These data match with the most productive authors and the countries where their institutes are located. Moreover, it is found that leading affiliations in antibiotic resistance and *Escherichia coli* studies in water environment are Universidade Regional do Cariri, Brazil, with 12 articles and USDA ARS Roman L. Hruska U.S. Meat Animal Research Center USA with 17 articles. Moreover, Universidad de La Rioja, Logrono, Spain, is listed on the top three author lists.

**Fig. 2:**
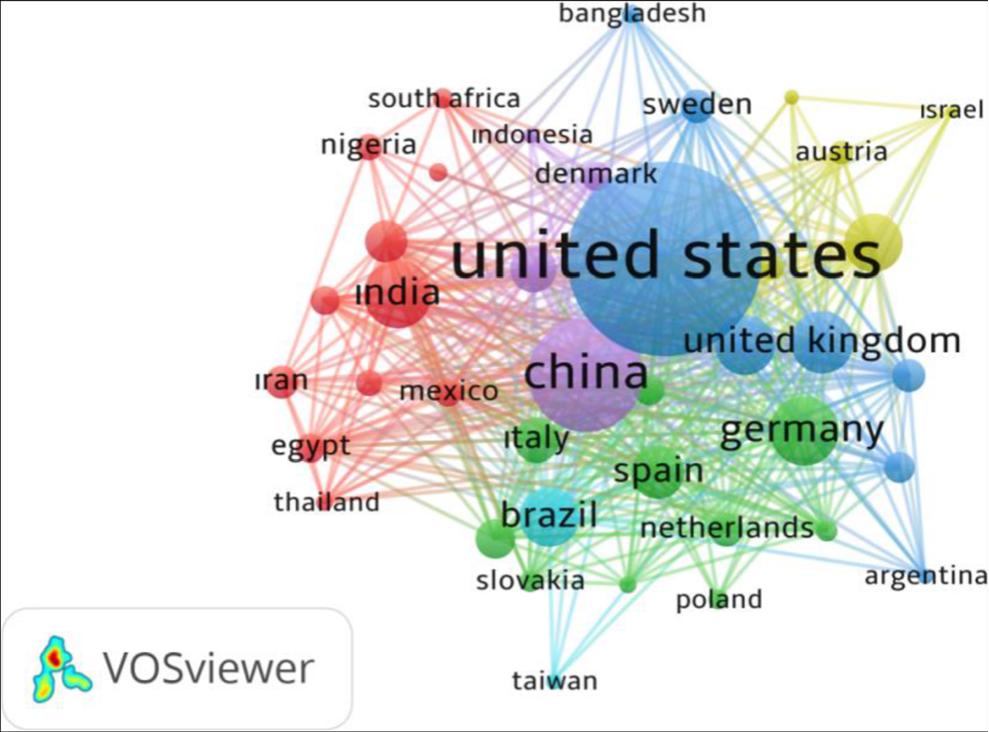
Network visualization map of countries for antibiotic resistance and *Escherichia coli* publications in the field of agricultural and biological sciences from 2015 to 2019 (the countries that appeared at least 10 times in the 1376 articles were included in the list)

**Fig. 3:**
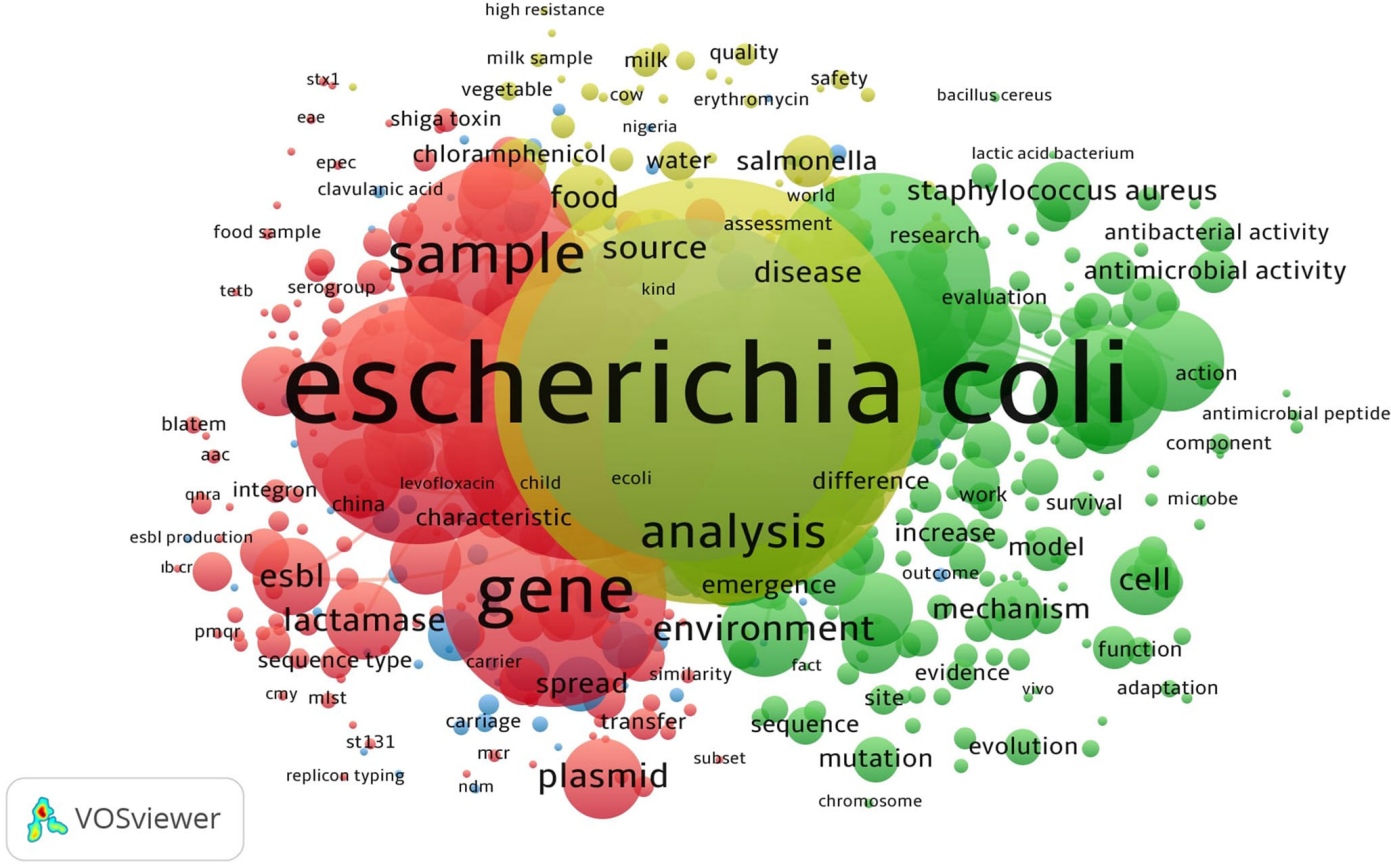
Research-topic network visualization for antibiotic resistance and *Escherichia coli* publications in the field of agricultural and biological sciences from 2015 to 2019 (the country names that appeared at least 15 times among 1376 articles were added in the list since the minimum occurrences number of a keyword for the analysis is 15)

The network visualization map of countries and the research-topic network map for antibiotic resistance and *Escherichia coli* publications in the field of Agricultural and Biological Sciences from 2015 to 2019 are given in Fig. 2 and Fig. 3, respectively. It is seen that words such as *Escherichia coli,* resistance, and gene are noticeable as a cluster. It is found that all these frequently used keywords are interrelated and compatible.

**Fig. 4:**
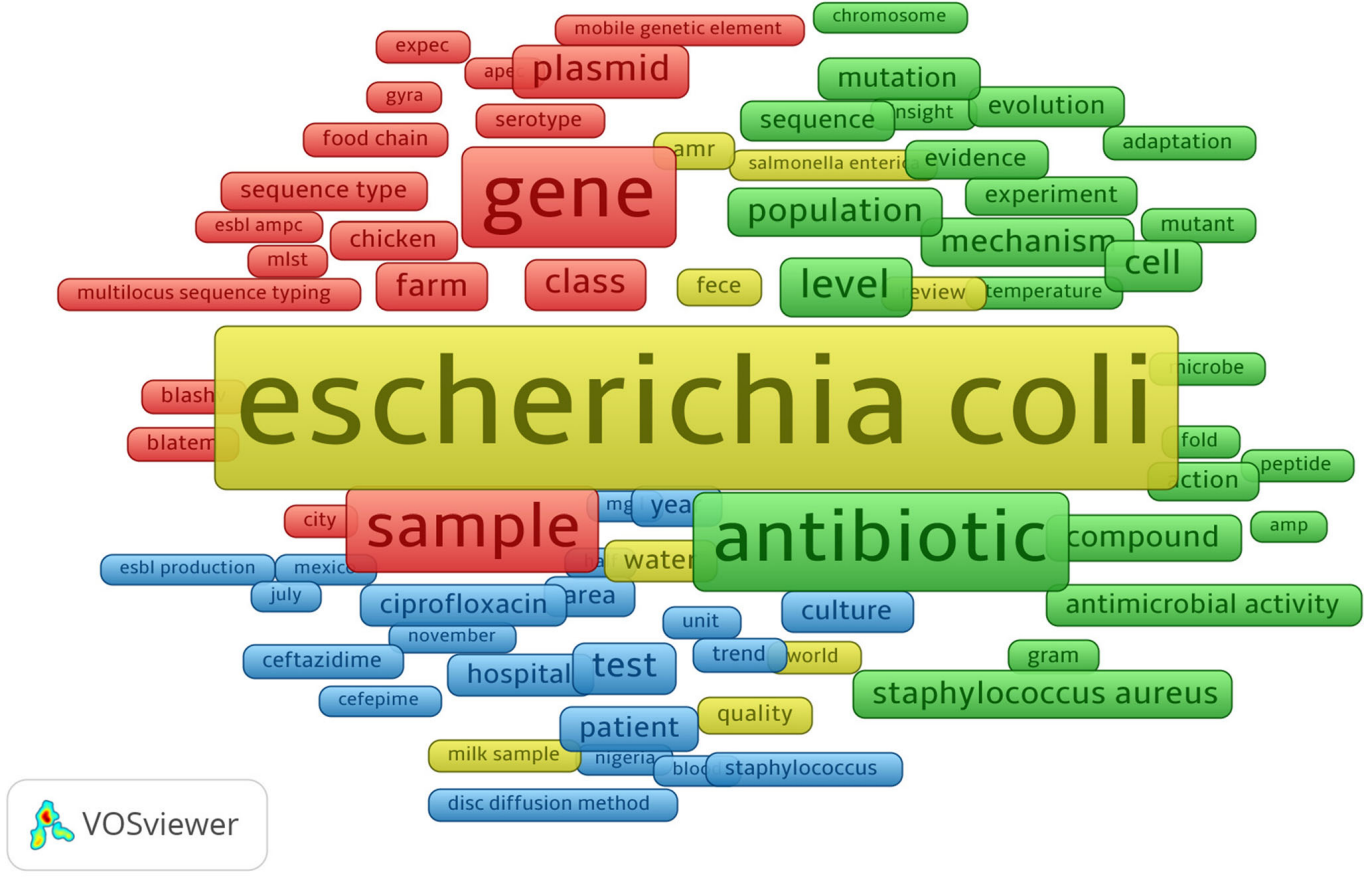
Research-topic density visualization map for antibiotic resistance and *Escherichia coli* publications in the field of Agricultural and Biological Sciences from 2015 to 2019 (the country names that appeared at least 10 times among 1376 articles were added in the list since the minimum occurrences number of a keyword for the analysis is 10)

Research-topic density visualization map for publications is also represented in Fig. 4. In total, 1376 articles on antibiotic resistance and *Escherichia coli* studies were retrieved in the field of agricultural and biological sciences from 2015 to 2019. Only 107 articles out of the 1376 articles were performed on antibiotic resistance and *Escherichia coli*, which cover only 7.77% of these studies. The drugs used were identified by using the titles, abstracts, and keywords in the bibliometric analysis in the field of agricultural and biological sciences.

From the results of the analysis, it is seen that multidrug is mostly preferred in antibiotic selection. The multidrug studies were expected to appear most often as *Escherichia coli* is resistant to many drugs. Therefore, 75% of the studies were multidrug studies which corresponds to our expectations.

**Fig. 5:**
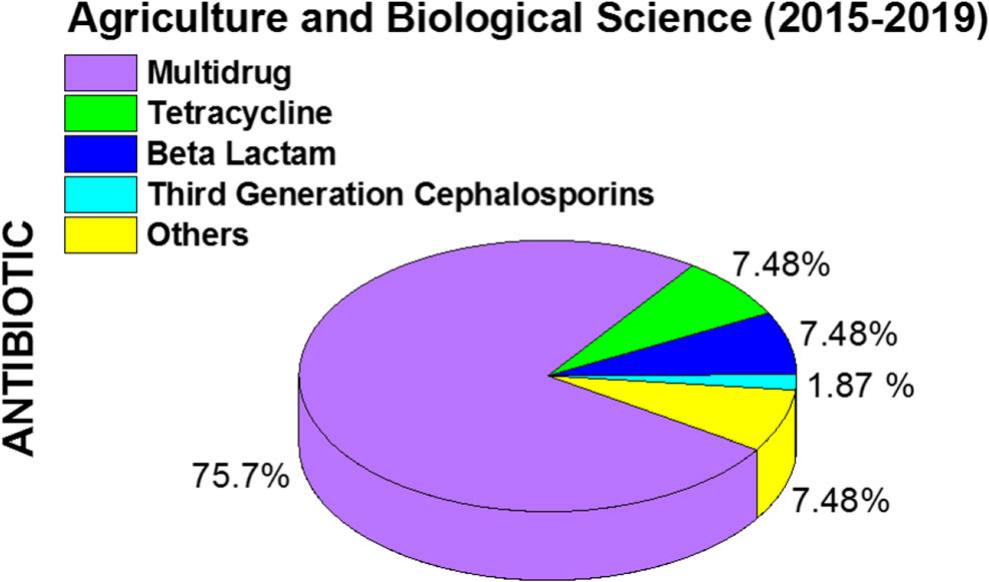
Percentage pie chart of antibiotics in the aquatic environment among articles on antibiotic resistance and *Escherichia coli* from 2015 to 2019 (others are aminoglycosides, ansamycins, carbacephem, carbapenems, cephalosporins (first, second, fourth, fifth generation), glycopeptides, lincosamides, macrolides, monobactams, oxazolidinones (Bs), penicillins, polypeptides, quinolones/fluoroquinolones, and sulfonamides)

As can be seen in Fig. 5, it is categorized into antibiotic categories multidrug, tetracycline, beta-lactam, cephalosporins (third generation), and others. The studies on beta-lactam and tetracycline (aminoglycosides, ansamycins, carbapenem, carbapenems, cephalosporins (first, second, fourth, fifth generation)), glycopeptides, lincosamides, macrolides, monobactams, oxazolidinones (Bs), penicillins, polypeptides, quinolones/fluoroquinolones, and sulfonamides are found to be two times greater than that of all antibiotics. Although cephalosporin is found to be of 1.87%, it is categorized as third generation as *Escherichia coli* is closely associated with cephalosporins (third generation) resistance. However, it was observed that there was less focus on cephalosporin resistance compared to the resistance to other antibiotics. It was seen that these publications mostly focused on tetracycline, beta-lactam, and multidrug.

The keywords of “antibiotic resistance” and “bibliometric” were used to discover the bibliometric analysis of antibiotic resistance using the Scopus search engine. As a result of this search, 29 studies were found. This bibliometric study compares the results of the bibliometric studies previously conducted in similar fields and provides information on the methods, antibiotics, resistance mechanisms, and examination methods used in the analysis. The first study on antibiotic resistance was conducted in 1999 and reported the possible solutions while emphasizing the economic harm of antibiotic resistance (García-Altés et al. 1999). In another study, by Washer and Joffe, a bibliometric analysis on methicillin-resistant *Staphylococcus aureus* in hospitals from 1995 to 2005 was performed by using data from newspaper articles, in which they reported inadequate hygiene in hospitals and health management errors (Washer and Joffe 2006). Another study in 2007 showed that the method evolved in a more technological direction by obtaining data from Medline and Embase (Matthys et al. 2007). Moreover, another bibliometric study in *Infection* journal from 1995 to 2011 stated that the most frequently studied subject in the journal was antibiotic resistance (Suárez 2012). However, the journal *Infection* was not found in the bibliometric analysis that we conducted on antibiotic resistance and *Escherichia coli* publications from 2015 to 2019. Here, it can be assumed that the journal *Infection* shifted its studies in a different direction or that antibiotic resistance on *Escherichia coli* was not studied. In 2013, a bibliometric study on rational drug use in Iran (which was searched on PubMed, Web of Science, Google Scholar, CINAHL, Proquest, International Pharmaceutical Abstract and Persian databases including SID, Iran Medex, and MagIran) was carried out using Web-based searches (Mousavi et al. 2013). Researchers performed another bibliometric analysis using PubMed from 1973 to 2013 in the evolution of the bacterial resistance of antibiotics in Mexico in 2014. *Salmonella* sp. bacteria from the class Enterobacteriaceae and beta-lactamase resistance were studied (Rodríguez-Noriega et al. 2013).

The most frequently reported resistant bacteria are found to be *Escherichia coli*, *Klebsiella pneumonia*, *Staphylococcus aureus*, and *Streptococcus pneumoniae*, followed by *Salmonella* spp. Multidrug resistance in *Escherichia coli* has become an alarming issue (WHO 2017; Poirel et al. 2018). *Escherichia coli* is sensitive to almost all antimicrobial agents. This strain of bacteria can accumulate resistance genes, mostly through horizontal gene transfer suggesting that researchers should focus on more *Escherichia coli* studies (Poirel et al. 2018).

A bibliometric analysis was carried out on methicillin-resistant *Staphylococcus aureus* using Web of Science (WoS) from 1961 to 2007. In the study, they stated that the leading country was the USA followed by the UK and Japan (Addicks et al. 2014). In addition, the USA took its first place as the most productive country in 2019. Although the databases used vary, it was observed that the information such as the number of publications, the most productive countries, institutes, the most productive writers, and featured journals that are provided by the bibliometric analysis have not changed. In 2016, another bibliometric analysis was performed on carbapenem resistance from 1986 to 2015 by using the Scopus database and the USA and China were reported to be the leading countries (Sweileh et al. 2016). Until 2016, the Scopus database was not found in bibliometric studies on antibiotic resistance. Scopus is a search engine that is reviewed by an independent review board and uses rich metadata architecture to link content, indexes, published ideas, and institutions from publishers. Thus, it gives the opportunity to examine the studies faster. In 2018, another bibliometric study on the antibiotics in aquatic systems was carried out from 1945 to 2017, providing comparison data to this study. The present study is considered the expansion of the 2018 study as well as including the data from 2015 to 2019 (Zheng et al. 2018). By taking advantage of these two studies, it can be said that the USA and China maintain their role as the leading countries in research output. The other result reported in 2019 is that the studies have mostly focused on wastewater in aquatic environments since 1945. The Scopus database was selected for the present study, and the Web of Science database was chosen in 2014. There is another study entitled as bibliometric analysis of global research on clavulanic acid and authors used Scopus in their analysis for a time span of 43 years (1975–2017) (Ramirez-Malule 2018).

Results from different publications provide information on antibiotics. As a result of excessive use of antibiotics, many antibiotics and residues were detected in water environments such as rivers, lakes, and seas (Hamscher et al. 2002). Studies reveal that even small amounts of antibiotics can cause the formation of antibiotic resistance genes (Costanzo et al. 2005; Kümmerer 2009; Watkinson et al. 2009). This interaction leads to the rapid spread of antibiotics since humans interact perpetually with the environment. Given all the reasons above, more efforts should be made to increase the number of studies and cooperation to tackle this global health problem.

## Conclusion

Antibiotics have become a global threat with the emergence of the resistance problem even though they play a significant role in improving human life quality. Given that, WHO has been supporting countries in the implementation of surveillance of antimicrobial consumption since 2016. WHO collected data on antibiotic use from 65 countries in 2015 and published a report covering from 2016 to 2018 (WHO 2020a). Thus, this cooperation between countries and number of studies should be increased to create a global response to globally threatening antibiotic resistance in this way. In Turkey, an informational meeting named “Rational use of antibiotics” was held in 2018; it was reported that antibiotic sales in 2011 constituted 13% of drug sales and this rate decreased to 10% after restrictions on their sale were imposed (Republic of Turkey Ministry of Health 2018). We found that 1376 articles on antibiotic resistance and *Escherichia coli* were published from 2015 to 2019 in the field of agricultural and biological sciences. The research emphasizes on *Escherichia coli*, a critical bacterium as they are listed as priority on the WHO priority list. The USA, China, and India are found to be the leading countries in terms of the number of published studies. As a result, to reduce and eliminate the antibiotic resistance, which constitutes a major problem globally, scientific cooperation should be strengthened, and more antibacterial treatment studies should be performed. Researchers will be able to use the right keywords, which are frequently used in the network visualization of the research subject, enabling them to find the most relevant publications.

## Future perspectives

COVID-19 has become a global problem and affected the entire world in 2020. The immune system should be maintained strong to be able to defeat the virus. According to one publication, during the incubation period and non-severe stages, a specific adaptive immune response is required to eliminate the virus and halt the disease progression to the severe stages (Shi et al. 2020). During the COVID-19 pandemic, antimicrobial resistance will potentially be empowered as antimicrobial activities have been drastically increased. Reducing the use of antimicrobial medicine and appropriate prescription along with correct treatment, reduction and preventing the infections could optimize antimicrobial activities. It is known that antibiotics are given to patients showing mild symptoms without having pneumonia or moderate symptoms with pneumonia (WHO 2020a, b). For this reason, the demand for antibiotics has increased during the pandemic problem that is experienced all over the world; therefore, the treatment becomes more difficult and presents a greater threat to patient's health. The development of new drug combinations and non-resistant applications will be an important step to overcome the issue in global human health.

## Supplementary Information

ESM 1 (DOCX 29 kb)

## Data Availability

Not applicable.
