# Supplementary material for: A comprehensive bibliometric overview: antibiotic resistance and Escherichia coli in natural water
Source: Environ Sci Pollut Res Int. 2021 May 6;28(25):32256–63. doi: 10.1007/s11356-021-14084-1 (PMC8102055; doi:10.1007/s11356-021-14084-1)
Supplement: Supplementary file 1 — (DOCX 29 kb) [file 11356_2021_14084_MOESM1_ESM.docx]

**Supplements**

**Table S1.** Water environment studies relevant to antibiotic resistance and *Escherichia coli* in the field of Agricultural and Biological Sciences in 2019.

| IN 2019 WATER ENVIRONMENT STUDIES | | | | |
| --- | --- | --- | --- | --- |
| COUNTRY | ANTIBIOTIC | ENVIRONMENT | JOURNAL'S NAME | QUARTILE OF  CATEGORY |
| United States | Beta lactam | Water, Faeces | Applied and environmental microbiology | Q1 |
| Austria | Beta lactam, carbapenem | Wastewater | Bodenkultur | Q4 |
| Canada | Multidrug | Water, Animal | Journal of Wildlife Diseases | Q2 |
| Mexico | Multidrug | Fresh water | International Journal of Food Microbiology | Q1 |
| Japan, China | Beta lactamase, carbapenemase, macrolide | Wastewater | Environmental Microbiology Reports | Q1 |
| India | Multidrug | Wastewater | Proceedings of the National Academy of Sciences India Section B - Biological Sciences | Q3 |
| United States | Multidrug | Fresh water | Molecular Ecology | Q1 |
| Poland | Imipenem, cefoxitin | Fresh water | Water (Switzerland) | Q1 |
| India | Amoxicillin, ampicillin, methicillin, penicillin | Sea water | International Journal of Environmental Science and Technology | Q2 |
| China | beta lactam, tetracyline, sulfonamide | Fresh water | Journal of Food Protection | Q2 |
| United States | Cefotaxime, clindamycin, sulfamethoxazole, tetracycline, vancomycin | Fresh water | International Journal of Food Microbiology | Q1 |
| China | Tetracycline | Sea water | Marine Biotechnology | Q1 |
| Poland | Ampicillin, imipenem, fosfomycin, meropenem | Fresh water | International Journal of Environmental Science and Technology | Q2 |
| United States | Multidrug | Fresh water | Applied and environmental microbiology | Q1 |
| Canada | Multidrug | Wastewater | PLoS ONE | Q1 |
| United States | Multidrug | Fresh water | Aquatic Mammals | Q3 |
| Malaysia | Multidrug | Wastewater, Animal, Soil | American Journal of Animal and Veterinary Sciences | Q3 |
| Bolivia | Beta lactam | Fresh water | PLoS ONE | Q1 |
| Indonesia | Multidrug | Sea water | AACL Bioflux | Q3 |

**Table S2** Water environment studies relevant to antibiotic resistance and *Escherichia coli* in the field of Agricultural and Biological Sciences in 2018.

| IN 2018 WATER ENVIRONMENT STUDIES | | | | |
| --- | --- | --- | --- | --- |
| COUNTRY | ANTIBIOTIC | ENVIRONMENT | JOURNAL'S NAME | QUARTILE OF  CATEGORY |
| Turkey | Methicillin | Water | Journal of the Textile Institute | Q2 |
| Bangladesh | Multidrug | Water | PLoS ONE | Q1 |
| Switzerland | Beta lactam, vancomycin | irrigation water | PLoS ONE | Q1 |
| South America | Multidrug | Sea water | Marine Pollution Bulletin | Q1 |
| Singapore | Multidrug | Sea water, Wastewater | Marine Pollution Bulletin | Q1 |
| China | Multidrug | Wastewater | Water (Switzerland) | Q1 |
| United States | Erythromycin, ampicillin, streptomycin, amoxicillin | irrigation water | Journal fur Verbraucherschutz und Lebensmittelsicherheit | Q3 |
| United States | Multidrug | Wastewater | Water (Switzerland) | Q1 |
| United States | Multidrug | Fresh water | PLoS ONE | Q1 |
| United States | Multidrug | Wastewater | Applied and Environmental Microbiology | Q1 |
| Philippines | Beta lactam, tetracycline | irrigation water (Water) | Water (Switzerland) | Q1 |
| United Kingdom | Multidrug | Sea water | Marine Pollution Bulletin | Q1 |
| United Kingdom | Multidrug | Fresh water, Wastewater | ISME Journal | Q1 |
| Peru | Multidrug | Fresh water, Wastewater, Seawater | Revista Peruana de Biologia | Q3 |
| United States | Multidrug | Sea water | Microbial Ecology | Q1 |
| China | Tetracycline | Fresh water, Animal | Chinese Journal of Applied Ecology | Q3 |
| Nigeria | Multidrug | irrigation water and manure | Journal of the Science of Food and Agriculture | Q1 |

**Table S3** Water environment studies relevant to antibiotic resistance and *Escherichia coli* in the field of Agricultural and Biological Sciences in 2017.

| IN 2017 WATER ENVIRONMENT STUDIES | | | | |
| --- | --- | --- | --- | --- |
| COUNTRY | ANTIBIOTIC | ENVIRONMENT | JOURNAL'S NAME | QUARTILE OF  CATEGORY |
| Sweden | Multidrug | Fresh water | PLoS ONE | Q1 |
| Viet Nam | Multidrug | Wastewater | Animal Health Research Reviews | Q1 |
| Bangladesh | Multidrug | Fresh water | PLoS ONE | Q1 |
| Tunisia | Multidrug | Seawater | Journal of Food Protection | Q2 |
| Norway | Multidrug | Sea water, Wastewater, Fresh water | PLoS ONE | Q1 |
| Portugal | Multidrug | Fresh water | International Journal of Food Microbiology | Q1 |
| South Africa | Imipenem, penicillin G | Wastewater , Freshwater | Water (Switzerland) | Q1 |
| India | Multidrug | Wastewater | PLoS ONE | Q1 |
| Philippines | Multidrug | irrigation waters | Journal of Environmental Science and Health - Part B Pesticides, Food Contaminants, and Agricultural Wastes | Q3 |
| United States | Ampicillin | Fresh water | International Journal of Food Microbiology | Q1 |
| China | Oxytetracycline | [Wastewater](https://www.scopus.com/sourceid/130078?origin=recordpage) | Chinese Journal of Applied and Environmental Biology | Q4 |
| United States | Multidrug | Fresh water, Waste water | pplied and Environmental Microbiology | Q1 |
| Spain | Multidrug | Fresh water | PLoS ONE | Q1 |
| Indonesia | Methicillin | Sea water | Biodiversitas | Q3 |
| Guadeloupe | Multidrug | Sea water, Wastewater, Fresh water | PLoS ONE | Q1 |
| Brazil | Beta lactam | Fresh water | Journal of Food Protection | Q2 |
| Poland | Multidrug | Fresh water | International Journal of Environmental Science and Technology | Q2 |
| South Africa | Multidrug | irrigation water | International Journal of Food Microbiology | Q1 |
| Thailand | Multidrug | Wastewater | PeerJ | Q1 |
| Japan | Multidrug | Fresh water | Applied and Environmental Microbiology | Q1 |
| Saudi Arabia | Multidrug | Wastewater | Applied and Environmental Microbiology | [Q1](https://www.scopus.com/sourceid/19618?origin=recordpage) |
| Thailand | Multidrug | Fresh water, Wastewater | Microbes and Environments | Q1 |
| Australia | Multidrug | Fresh water | PLoS ONE | Q1 |
| Bangladesh | Multidrug | Fresh water, Wastewater | Applied and Environmental Microbiology | Q1 |
| Nigeria | Multidrug | Fresh water | Annual Research and Review in Biology | Q3 |
| Nigeria | Multidrug | Fresh water, Soil, Wastewater | Annual Research and Review in Biology | Q3 |

**Table S4** Water environment studies relevant to antibiotic resistance and *Escherichia coli* in the field of Agricultural and Biological Sciences in 2016.

| IN 2016 WATER ENVIRONMENT STUDIES | | | | |
| --- | --- | --- | --- | --- |
| COUNTRY | ANTIBIOTIC | ENVIRONMENT | JOURNAL'S NAME | QUARTILE OF  CATEGORY |
| South Africa | Multidrug | Irrigation water | Journal of Food Protection | Q2 |
| United States | Multidrug | Fresh water | Microbial Ecology | Q1 |
| Austria | Multidrug | Fresh water | PLoS ONE | Q1 |
| Germany | Multidrug | Wastewater , Freshwater | Microbial Ecology | Q1 |
| Ethiopia | Multidrug | Fresh water | PLoS ONE | [Q1](https://www.scopus.com/sourceid/10600153309?origin=recordpage) |
| France | Multidrug | Wastewater | Environmental Microbiology Reports | Q1 |
| India | Multidrug | Wastewater | Journal of Biosciences | Q1 |
| United States | Multidrug | Sea water, Fresh water | Journal of Aquatic Animal Health | Q3 |
| China | Multidrug | Fresh water, Waste water | PLoS ONE | Q1 |
| Chile | Multidrug | Sea water, Wastewater | Polar Science | Q2 |
|  | Multidrug | Wastewater | Indian Journal of Ecology | Q4 |
| Canada | Multidrug | Wastewater | PLoS ONE | [Q1](https://www.scopus.com/sourceid/10600153309?origin=recordpage) |
| Russian Federation, Germany | Multidrug | Wastewater | International Journal of Environmental Science and Technology | Q2 |
| Canada | Multidrug | Fresh water | Applied and Environmental Microbiology | Q1 |
| United States | Multidrug | Fresh water | Water (Switzerland) | Q1 |
| India | Multidrug | Fresh water | PeerJ | Q1 |
| Sweden | Multidrug | Wastewater | Applied and Environmental Microbiology | Q1 |
| India | Multidrug | Wastewater | Regional Studies in Marine Science | Q2 |
| United States | Multidrug | Fresh water, Sea water, Wastewater | Applied and Environmental Microbiology | Q1 |
| Iran | Multidrug | Sea water | Russian Journal of Marine Biology | Q3 |
| Nigeria | Multidrug | Wastewater | Quality Assurance and Safety of Crops and Foods | Q3 |
| Brazil | Multidrug | Fresh water | Revista Ambiente e Agua | Q3 |

**Table S5** Water environment studies relevant to antibiotic resistance and *Escherichia coli* in the field of Agricultural and Biological Sciences in 2015.

| IN 2015 WATER ENVIRONMENT STUDIES | | | | |
| --- | --- | --- | --- | --- |
| COUNTRY | ANTIBIOTIC | ENVIRONMENT | JOURNAL'S NAME | QUARTILE OF  CATEGORY |
| Japan | Multidrug | Sea water | Microbes and Environments | Q1 |
| Bangladesh | Penicillin , vancomycin, rifampicin | Wastewater | Bangladesh Journal of Botany | Q4 |
| Singapore | Chloramphenicol, florfenicol, sulfonamide, trimethoprim | Sea water | PLoS ONE | Q1 |
| Nigeria | Multidrug | Wastewater | Environmental Technology and Innovation | Q2 |
| Japan | Cefotaxime, imipenem, streptomycin | Wastewater | PLoS ONE | Q1 |
| Netherlands | Multidrug | Wastewater, Fresh water, Soil | PLoS ONE | Q1 |
| United States | Multidrug | Wastewater, Fresh water | PLoS ONE | Q1 |
| Philippines | Amoxicillin | Fresh water | Advances in Environmental Biology | Q4 |
| Tunisia | Multidrug | Wastewater | International Journal of Food Microbiology | Q1 |
| Netherlands | Multidrug | Wastewater | PLoS ONE | Q1 |
| Tanzania | Tetracycline, ampicillin | Wastewater | Preventive Veterinary Medicine | Q1 |
| Australia | Multidrug | Sea water | Journal of Wildlife Diseases | Q2 |
| Botswana | Multidrug | Wastewater | Journal of Wildlife Diseases | Q2 |
| Slovakia | Multidrug | Wastewater | Annals of Agricultural and Environmental Medicine | Q2 |
| South Korea | Multidrug | Wastewater | Applied and Environmental Microbiology | Q1 |
| United States | Multidrug | Wastewater, Food, Faeces | Journal of Food Science | Q1 |
| Turkey | Multidrug | Sea water | Diseases of Aquatic Organisms | Q2 |
| United States | Tetracycline, florfenicol | Sea water | Environmental Microbiology Reports | Q1 |
| Canada | Multidrug | Fresh water | Foodborne Pathogens and Disease | Q1 |
| South Africa | Multidrug | Fresh water | Journal of Food Protection | Q2 |
| Brazil | Multidrug | Sea water | Boletim do Instituto de Pesca | Q3 |
| United States | Multidrug | Fresh water | Applied and Environmental Microbiology | Q1 |
| Croatia | Multidrug | Sea water | Marine Pollution Bulletin | Q1 |
